# Identification and validation of FGFR2 peptide for detection of early Barrett's neoplasia

**DOI:** 10.18632/oncotarget.19764

**Published:** 2017-08-01

**Authors:** Juan Zhou, Lei He, Zhijun Pang, Henry D. Appelman, Rork Kuick, David G. Beer, Meng Li, Thomas D. Wang

**Affiliations:** ^1^ Department of Medicine, Division of Gastroenterology, University of Michigan, Ann Arbor, Michigan 48109, USA; ^2^ Biotechnology Center, School of Pharmacy, The Fourth Military Medical University, Xi’an 710032, China; ^3^ Department of Pathology, University of Michigan, Ann Arbor, Michigan 48109, USA; ^4^ Department of Biostatistics, University of Michigan, Ann Arbor, Michigan 48109, USA; ^5^ Department of Surgery, Section of Thoracic Surgery, University of Michigan, Ann Arbor, Michigan 48109, USA; ^6^ Department of Biomedical Engineering, University of Michigan, Ann Arbor, Michigan 48109, USA; ^7^ Department of Mechanical Engineering, University of Michigan, Ann Arbor, Michigan 48109, USA

**Keywords:** FGFR2, peptide, esophagus, cancer

## Abstract

The incidence of esophageal adenocarcinoma (EAC) is rising rapidly, and early detection within the precursor state of Barrett's esophagus (BE) is challenged by flat premalignant lesions that are difficult detect with conventional endoscopic surveillance. Overexpression of cell surface fibroblast growth factor receptor 2 (FGFR2) is an early event in progression of BE to EAC, and is a promising imaging target. We used phage display to identify the peptide SRRPASFRTARE that binds specifically to the extracellular domain of FGFR2. We labeled this peptide with a near-infrared fluorophore Cy5.5, and validated the specific binding to FGFR2 overexpressed in cells *in vitro*. We found high affinity k_d_ = 68 nM and rapid binding k = 0.16 min^−1^ (6.2 min). In human esophageal specimens, we found significantly greater peptide binding to high-grade dysplasia (HGD) versus either BE or normal squamous epithelium, and good correlation with anti-FGFR2 antibody. We also observed significantly greater peptide binding to excised specimens of esophageal squamous cell carcinoma and gastric cancer compared to normal mucosa. These results demonstrate potential for this FGFR2 peptide to be used as a clinical imaging agent to guide tissue biopsy and improve methods for early detection of EAC and potentially other epithelial-derived cancers.

## INTRODUCTION

There are over 450,000 new cases of esophageal cancer diagnosed worldwide each year, resulting in more than 400,000 deaths annually [1]. Esophageal adenocarcinoma (EAC) represents the majority of cases in the U.S., where the incidence and mortality continue to rise rapidly [2]. This trend is attributed to increasing obesity and chronic gastroesophageal reflux disease (GERD) [3]. Barrett's esophagus (BE) is the replacement of normal squamous epithelium with intestinal metaplasia, and can transform into low-grade dysplasia (LGD) and progress to high-grade dysplasia (HGD) prior to developing EAC [4]. LGD represents increased risk, but pathological diagnosis of this condition can be subjective and inconsistent in interpretation [5]. Conventional white light endoscopy with random four-quadrant tissue biopsies has been recommended for surveillance of BE patients [6]. Therapy includes endoscopic mucosal resection (EMR), radio-frequency ablation (RFA), and surgery for improved patient outcomes [6]. Unfortunately, endoscopic strategies for detection of pre-malignant lesions are limited by sampling error, flat architecture, and patchy distribution [7]. Molecular changes associated with gene alterations precede histopathological abnormalities, and may be developed for imaging as an adjunct to endoscopy for early cancer detection [8].

Receptor tyrosine kinases (RTKs) are expressed on the cell membrane, where they are accessible for *in vivo* imaging [9]. They occupy key regulation points for cell signaling during cancer progression. FGFR2 has been found to be highly expressed early in progression from BE to EAC [10]. FGFR2 is a member of the fibroblast growth factor receptor (FGFR) family that includes FGFR1-4, [11] which are glycoproteins located on the cell surface, and consist of 3 extracellular immunoglobulin (Ig)-like domains, a hydrophobic transmembrane region, and a cytoplasmic domain that contains a tyrosine kinase catalytic domain [12]. More than 20 alternative splicing variants of FGFR2 have been identified [13]. Major splicing occurs in the carboxyl terminus of the third Ig-like domain (D3). Isoform IIIb or IIIc of FGFR2 is generated when the C-terminus of D3 is encoded by either exon 8 or 9, respectively. FGF-1, 3, 7, 10, and 22 are known to bind to FGFR2b, while FGF-1, 2, 4, 6, 9, 17, and 18 bind to FGFR2c. Binding of FGF to FGFR2 phosphorylates specific tyrosine residues that mediate interactions with cytosolic adaptor proteins and activates intracellular signaling cascades, such as RAS-MAPK, PI3K-AKT, PLCγ, and STAT [14–18].

Use of peptides to detect and localize Barrett's neoplasia with imaging has recently been demonstrated in the clinic [19, 20]. An empiric peptide labeled with FITC was administered topically to the mucosal surface. Early neoplasia was detected with 94% specificity and 96% positive predictive value. Included in the analysis were 28 flat lesions (Paris 0-IIb) that were poorly visualized with white light. Binding occurred within 5 min, which resulted in minimal time added to the diagnostic procedure. Peptides have high diversity, and can achieve high specificity with binding affinities on the nanomolar scale. This probe platform has flexibility to be labeled with a broad range of fluorophores, [21] and is inexpensive to produce in large quantities. These features of peptides are well suited for clinical use in high volume procedures. Barrett's metaplasia involves only a few centimeters of the distal esophagus, thus topical peptide administration can achieve high concentrations to maximize target interactions and achieve rapid binding with minimal risk for toxicity [22]. The quantity, hence cost, of the imaging agent needed is minimized, and probe biodistribution to non-target tissues is avoided for increased safety. Here, we aim to develop a novel peptide that targets FGFR2, and demonstrate specific binding to Barrett's neoplasia. In the future, this peptide can be used clinically for early cancer detection, image-guided resection, risk stratification, and monitoring of therapeutic efficacy.

## RESULTS

### Selection of peptide specific for FGFR2

We performed immunohistochemistry (IHC) on specimens of human esophagus, including squamous (SQ), Barrett's esophagus (BE), low-grade dysplasia (LGD), high-grade dysplasia (HGD), and esophageal adenocarcinoma (EAC), that were classified by an expert gastrointestinal pathologist (HDA) to demonstrate representative levels of FGFR2 expression, Supplementary Figure 1. The extra-cellular domain (ECD) of FGFR2 consists of a signal peptide (SP) and 3 extracellular immunoglobulin-like domains (D1-D3), Supplementary Figure 2A. We used FGFR2-ECD with purity >97% by HPLC. SDS-PAGE shows apparent molecular mass of ~65-75 kDa, Supplementary Figure 2B. This result is slightly higher than the expected value of 41 kDa as a result of glycosylation of the FGFR2 protein. After 4 rounds of biopanning with phage display, we found 2 sequences that showed enrichment. In 50 clones, SRRPASFRTARE appeared 15X and GLHTSATNLYLH appeared 4X. GLHTSATNLYLH was found previously when we biopanned against other protein targets, and is likely an unrelated sequence.

### Peptide specific for FGFR2

**Figure 1:**
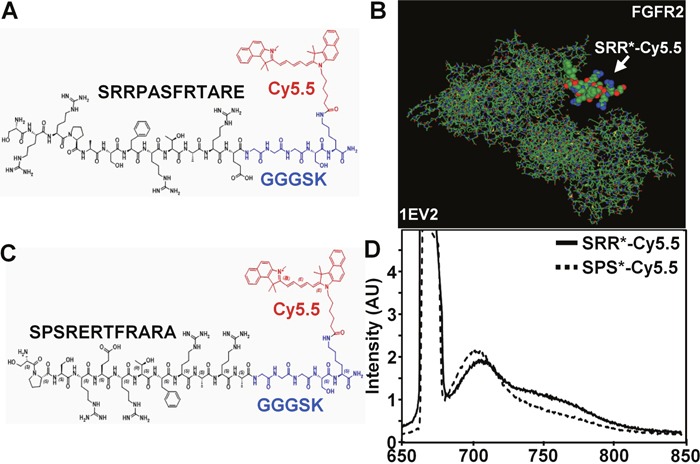
Peptide specific for FGFR2 Chemical structure is shown for 12 amino acid (aa) peptide sequence **(A)** SRRPASFRTARE (SRR^*^) found to be specific for FGFR2, and **(B)** scrambled peptide SPSRERTFRARA (SPS^*^) used for control. A Cy5.5 fluorophore (red) is attached via a GGGSK linker (blue) to prevent steric hindrance. **(C)** SRR^*^-Cy5.5 was found using a structural model (1EV2) to bind to the extracellular domain (ECD) of FGFR2c (147-366 aa) with E_t_ = −290.43 while SPS^*^-Cy5.5 resulted in E_t_ = −277.37. **(D)** Fluorescence spectra of SRR^*^-Cy5.5 and SPS^*^-Cy5.5 at 10 μM concentration in PBS with excitation at λ_ex_ = 671 nm shows peak emission at λ_em_ = 710 nm in the NIR spectrum.

We synthesized the 12 amino acid sequence SRRPASFRTARE (black) and attached the fluorophore Cy5.5 (red) via a GGGSK linker (blue) on the C-terminus, hereafter SRR^*^-Cy5.5, Figure 1A. Cy5.5 was chosen for photostability and high quantum yield in the near-infrared (NIR) spectrum [23]. We used a linker to prevent steric hindrance of the peptide by the dye. We then used a structural model (1EV2), [24] Figure 1B, and found SRR^*^-Cy5.5 to bind to domains D2 and first half of D3 of FGFR2- ECD with a total energy E_t_ = −290.43. This domain is the same in either isoform FGFR2IIIb or FGFR2IIIc. We also used this model to develop a scrambled sequence SPSRERTFRARA for a control, Figure 1C. This peptide was also labeled with Cy5.5 via a GGGSK linker, hereafter SPS^*^-Cy5.5. For SPS^*^-Cy5.5, we calculated E_t_ = −277.37. The fluorescence spectra of SRR^*^-Cy5.5 and SPS^*^-Cy5.5 with λ_ex_ = 671 nm excitation revealed a peak emission at λ_em_ = 710 nm, Figure 1D. We purified SRR^*^-Cy5.5 and SRS^*^-Cy5.5 to >97% on HPLC, and measured an experimental mass-to-charge (m/z) ratio on mass spectrometry of 2385.31 for both peptides that agreed with the expected value, Supplementary Figure 3.

### Confocal fluorescence microscopy

**Figure 2:**
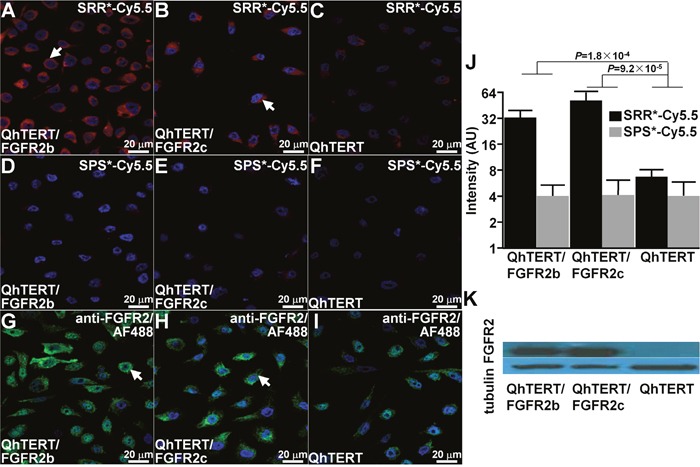
Validation of specific FGFR2 peptide binding to cells On confocal microscopy, we observed strong binding of SRR^*^-Cy5.5 (red) to surface of QhTERT cells that express **(A)** FGFR2b and **(B)** FGFR2c compared with **(C)** wild type. **(D-F)** Minimal signal is seen with the scrambled peptide SPS^*^-Cy5.5. **(G-I)** Strong binding is seen with anti-FGFR2 antibody labeled with AF488 (green) used as positive control. All experiments were performed in triplicate. **(J)** Quantified results show significantly higher mean fluorescence intensities for SRR^*^-Cy5.5 versus SPS^*^-Cy5.5 (control). We log-transformed and averaged measurements for 3 random cells on each of 3 slides per condition, and fit an ANOVA model with terms for 6 means. **(K)** Western blot shows protein expression level of FGFR2 for each cell.

On confocal microscopy, we validated specific peptide binding to human immortalized BE cells that express FGFR2. We observed strong signal with SRR^*^-Cy5.5 on the surface of QhTERT cells that express either FGFR2b or FGFR2c and minimal signal for wild-type, Figure 2A-2C. Minimal binding was observed for the scrambled control peptide SPS^*^-Cy5.5 with all cells, Figure 2D-2F. We confirmed these findings using anti-FGFR2 antibody labeled with AF488, Figure 2G-2I. We quantified our results, and found a significantly greater mean fluorescence intensity for SRR^*^-Cy5.5 than for control with QhTERT cells that express either FGFR2b or FGFR2c compared with wild-type, Figure 2J. Western blot shows of FGFR2 expression level for each cell, Figure 2K.

### Competition for peptide binding

**Figure 3:**
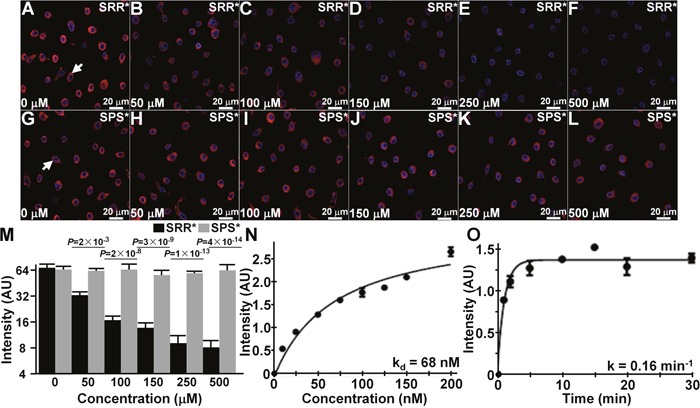
Characterization of specific FGFR2 peptide binding **(A-F)** On competition with addition of unlabeled SRR^*^ peptide at concentrations of 50 μM and higher, we observed a significant decrease in binding of SRR^*^-Cy5.5 to QhTERT cells that express FGFR2c. **(G-L)** Non-significant differences were found with the addition of unlabeled SPS^*^. **(M)** Fluorescence intensities were fit to an ANOVA model with terms for 12 means. Signal was quantified from an average of 3 cells chosen randomly from 3 slides for each condition. *P*-values are shown above data, and compare differences in intensity with addition of unlabeled SRR^*^ and SPS^*^ at each concentration with the same difference with no unlabeled peptide. **(N)** Using flow cytometry, we measured an apparent dissociation constant of k_d_ = 68 nM, R^2^= 0.96, and **(O)** an apparent association time constant of k = 0.16 min^−1^ (6.2 min) for binding of SRR^*^-Cy5.5 to QhTERT cells that express FGFR2c. These results are representative of 3 independent experiments.

We administered unlabeled SRR^*^, and used confocal microscopy to observe competition for binding of SRR^*^-Cy5.5 to QhTERT cells that express FGFR2c, Figure 3A-3L. We quantified the mean fluorescence intensities, and observed a significant reduction at concentrations of 50 μM and greater of SRR^*^ compared with that at 0 μM, Figure 3M. No significant difference was found with addition of unlabeled control SPS^*^ at any concentration. This result supports binding of the peptide rather than the fluorophore to FGFR2.

### Characterization of peptide binding

Using flow cytometry, we measured an apparent dissociation constant of k_d_ = 68 nM for binding of SRR^*^-Cy5.5 to QhTERT cells that express FGFR2c, Figure 3N. This result provides an estimate for binding affinity. We also measured an apparent association time constant of k = 0.16 min^−1^ for binding of SRR^*^-Cy5.5 to QhTERT cells that express FGFR2c, Figure 3O. This result provides time scale of ~6.2 min for onset of binding.

### Binding of FGFR2 peptide and antibody to human esophageal neoplasia

**Figure 4:**
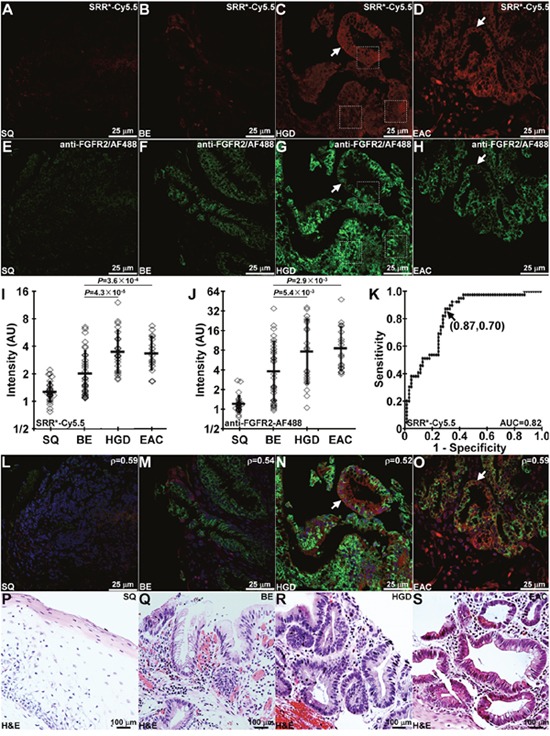
Binding of FGFR2 peptide to Barrett's neoplasia On representative images collected with confocal microscopy of human esophageal specimens *ex vivo*, SRR^*^-Cy5.5 (red) shows minimal staining to **(A)** squamous (SQ) and **(B)** Barrett's esophagus (BE) and strong binding (arrows) to **(C)** high-grade dysplasia (HGD) and **(D)** esophageal adenocarcinoma (EAC). **(E-H)** Anti-FGFR2 antibody labeled with AF488 (green) was used as a positive control, and shows weak staining to SQ and BE but strong binding (arrows) to HGD and EAC. We quantified the fluorescence intensities from the mean of a set of 3 boxes with dimensions of 30×30 μm^2^ placed over cells, shown in panels (C) and (G). From n = 28, 33, 22, and 17 specimens of SQ, BE, HGD, and EAC, respectively, we found significantly greater mean fluorescence intensity from HGD and EAC compared with that for BE with **(I)** SRR^*^-Cy5.5 and **(J)** AF488-labeled anti-FGFR2 using an ANOVA model with terms for 4 means on log-transformed data. **(K)** ROC curve shows 87% sensitivity and 70% specificity for detecting Barrett's neoplasia (HGD and EAC) at a T/B ratio of 3.0. **(L-O)** Merged images shows co-localization of peptide (red) and antibody (green) binding. We determined a Pearson's correlation coefficient of ρ = 0.59, 0.54, 0.52 and 0.59 for SQ, BE, HGD and EAC, respectively. Representative histology (H&E) are shown for **(P)** SQ, **(Q)** BE, **(R)** HGD, and **(S)** EAC.

On confocal microscopy, we evaluated staining of the FGFR2 peptide SRR^*^-Cy5.5 to sections of human esophagus *ex vivo*. We observed minimal fluorescence intensity with squamous (SQ) and BE, Figure 4A, 4B, and strong signal with HGD and EAC, Figure 4C, 4D. We confirmed these results with AF488-labeled anti-FGFR2 antibody, Figure 4E-4H. Fluorescence intensities were measured from a set of 3 boxes with dimensions of 30×30 μm^2^ to calculate the target-to-background (T/B) ratio. The mean (±std) T/B ratio for SRR^*^-Cy5.5 was significantly higher for HGD and EAC than that for BE and SQ, Figure 4I. These results are consistent with that with anti-FGFR2 antibody, Figure 4J. From the ROC curve, we found 87% sensitivity and 70% specificity for this peptide to detect Barrett's neoplasia (HGD and EAC) at a T/B ratio of 3.0 when compared with pathology, Figure 4K. We plot the fluorescence intensities for all specimens, and found good correlation between SRR^*^-Cy5.5 and anti-FGFR2-AF488 with R = 0.66, Supplementary Figure 4. Co-localization of peptide and antibody binding can be seen on merged images, Figure 4K-4N. Corresponding histology (H&E) were shown in Figure 4O-4R.

### Effect of FGFR2 peptide binding on cell signaling

**Figure 5:**
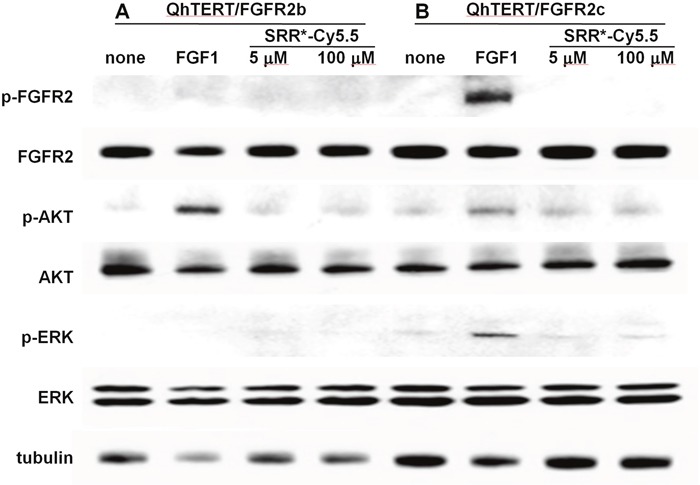
FGFR2 peptide does not affect cell signaling Western blot shows no obvious change in phosphorylation for either FGFR2 (p-FGFR) or downstream AKT (p-AKT) and ERK (p-ERK) with addition of SRR^*^ peptide at 5 and 100 μM to QhTERT cells that express FGFR2b or FGFR2c compared with untreated cells. Addition of FGF1 as positive control to bind FGFR2b and FGFR2c shows phosphorylation activity for FGFR2 (p-FGFR), downstream AKT (p-AKT) and ERK (p-ERK), especially in QhTERT cells expressing FGFR2c.

We evaluate the effect of peptide binding on downstream signaling in QhTERT cells that express either FGFR2b or FGFR2c. Western blot shows no change in phosphorylation of either FGFR2 (p-FGFR) or downstream AKT (p-AKT) and ERK (p-ERK) with addition of SRR^*^ peptide at a concentration of either 5 or 100 μM, Figure 5A. By comparison, we observed strong phosphorylation activity of FGFR2 (p-FGFR), downstream AKT (p-AKT) and ERK (p-ERK) with addition of positive control FGF1 in QhTERT cells that express FGFR2c and to some extent or FGFR2b.

### Binding of FGFR2 peptide to human squamous cell and gastric cancer

On confocal microscopy, we observed strong fluorescence intensity from incubation of the FGFR2 peptide SRR^*^-Cy5.5 to sections of human esophageal squamous cell cancer (SCC) *ex vivo* in n = 35 patients, Supplementary Figure 5A. We confirmed this result with AF488-labeled anti-FGFR2 antibody, Supplementary Figure 5B. We observed good co-localization of peptide and antibody binding on merged images, Supplementary Figure 5C. Representative histology (H&E) for SCC is shown, Supplementary Figure 5D. By comparison, we observed minimal fluorescence intensity in normal human esophagus with either peptide or antibody, Supplementary Figure 5E-5G. Representative histology (H&E) for normal stomach is shown, Supplementary Figure 5H.

On confocal microscopy, we also observed strong fluorescence intensity from staining of the FGFR2 peptide SRR^*^-Cy5.5 to sections of human gastric cancer *ex vivo* in n = 33 patients, Supplementary Figure 6A. We confirmed this result with AF488-labeled anti-FGFR2 antibody, Supplementary Figure 6B. We observed good co-localization of peptide and antibody binding on merged images, Supplementary Figure 6C. Representative histology (H&E) for gastric cancer is shown, Supplementary Figure 6D. By comparison, we observed minimal fluorescence intensity in normal human stomach with either peptide or antibody, Supplementary Figure 6E-6G. Representative histology (H&E) for normal stomach is shown, Supplementary Figure 6H.

We quantified fluorescence intensities from a set of 3 boxes with dimensions of 30×30 μm^2^ in each image and found a significantly greater result for SCC versus normal and for gastric cancer versus normal, Supplementary Figure 6I, 6J, respectively.

## DISCUSSION

Here, we have identified a novel peptide specific for FGFR2 that binds to the extra-cellular Ig-like domain of isoforms IIIb and IIIc. Expression of FGFR2 has been identified as an early event in progression from BE to EAC [10]. We demonstrate accessibility for imaging by showing that this peptide binds to the cell membrane *in vitro*, and confirm specificity for FGFR2 using siRNA knockdown and competition results. These studies were rigorously controlled using a scrambled peptide. We found this peptide to bind cells with high affinity of k_d_ = 68 nM and rapid binding onset of k = 0.16 min^−1^ (6.2 min). We labeled this peptide with Cy5.5, a NIR fluorophore, and visualized specific cell surface staining to neoplasia in human specimens of BE, SCC, and gastric cancer *ex vivo*. These findings were confirmed using a known anti-FGFR2 antibody. We also provide evidence that peptide binding does not affect downstream cell signaling. This peptide can potentially be used for therapy by labeling nanocarriers to achieve site-specific drug delivery of high payloads [25]. These results justify further development of this peptide for clinical imaging in patients at high risk for epithelial-derived cancers in the esophagus and stomach.

Peptides are being developed for early detection of Barrett's neoplasia. Wide-field fluorescence imaging techniques have been demonstrated that rapidly visualize large mucosal surfaces to provide a “red flag” region to identify high risk areas and guide tissue resection [20]. We have previously identified a 7 amino acid peptide ASYNYDA that was labeled with FITC [19]. This sequence was selected using phage display in an unbiased screen against human H460 adenocarcinoma cells found later to be lung rather than esophageal in origin, [26] and the protein target was not definitively determined. We have also identified peptides specific for EGFR and ErbB2. [27, 28] These genes are frequently highly amplified in EAC [29]. A panel of targets may be needed to achieve acceptable performance for early detection of Barrett's neoplasia. [10] Peptides have similar binding onsets, and multiplexed detection has been demonstrated *in vivo* [21]. Previously, a peptide specific for FGFR2 was developed as a precursor for red luminescent gold nanoclusters [30]. This peptide binds to human esophageal SCC cells *in vitro* and produces good luminescence with high stability, non-toxicity and biocompatibility. Development for clinical imaging has not been performed.

New imaging strategies are needed for early detection of Barrett's neoplasia. Probes that target FGFR2, including antibodies, lectins, and small molecules, are being developed. GP369 is an antibody specific for FGFR2b that exhibits potent anti-tumor activity [31]. Antibodies have been repurposed for *in vivo* imaging, however widespread clinical use of this probe platform for diagnostics has been limited by slow binding kinetics, immunogenicity, and high production costs [32]. Lectins have been shown to target Barrett's neoplasia *ex vivo* [33]. However, these agents have low diversity and may not achieve sufficient binding affinity for *in vivo* use. Moreover, the glycoprotein targets are under expressed with progression of disease, thus produce a negative contrast that can be prone to false-positives *in vivo*. Tyrosine kinase inhibitors have been shown to decrease survival of gastric cancer cells with FGFR2 amplifications *in vitro* [34]. Other methods of wide-area endoscopy, including chromoendoscopy, [35] narrowband imaging (NBI), [36] and autofluorescence imaging (AFI), [37] have been evaluated clinically, but provide low intrinsic contrast and are based on non-specific mechanisms. In clinical studies, these approaches have not demonstrated a clear advantage over conventional white light endoscopy with random biopsies.

In addition to Barrett's neoplasia, FGFR2 is overexpressed in other epithelial-derived cancers, including esophageal SCC, [38] gastric, [39] esophagogastric junction, [40] colorectal, [41] pancreatic, [42] and breast [43]. We present immunofluorescence results to support broad use of this FGFR2 peptide for detection of esophageal SCC and gastric cancer, Supplementary Figures 5, 6. In Barrett's neoplasia, we found LGD and HGD to be more difficult to distinguish than esophageal SCC because BE has higher FGFR2 expression than normal squamous epithelium. For future clinical validation, we will use current Good Manufacturing Practices (cGMP) to synthesize the FGFR2 peptide for use in a rigorous animal pharmacology/toxicology study to be performed with Good Laboratory Practices (GLP). This data will be included in an Investigational New Drug (IND) application submitted to the FDA for *in vivo* use in human subjects. Because the region of the distal esophagus affected by BE is only a few centimeters in length, the peptide can be administered topically rather than intravenously at a high concentration to maximize target interactions and achieve rapid binding with minimal risk for toxicity [20]. In conclusion, expression of FGFR2 is an early event in progression from BE to EAC. We have identified and validated a peptide specific for this cell surface target for future clinical use in early detection of EAC. This strategy may be applied to imaging of other epithelial-derived cancers.

## MATERIALS AND METHODS

### Tissues, cells, and chemicals

All human esophageal specimens were obtained with written, informed patient consent per approval and guidelines of the University of Michigan Institutional Review Board (IRB). Human non-dysplastic Barrett's esophagus (BE) cells immortalized with hTert (QhTERT) were obtained from the American Type Culture Collection (ATCC) and cultured in keratinocyte-serum free medium containing bovine pituitary extract and human recombinant EGF (ThermoFisher #17005042). QhTERT cells with stable expression of FGFR2b or FGFR2c were provided (DGB) [44]. We cultured these cells with keratinocyte-serum free medium containing bovine pituitary extract and human recombinant EGF (ThermoFisher #17005042) and added 1 μg/mL of puromycin-dihydrochloride (Invitrogen #A11138-03). All cells were cultured at 37°C in 5% CO_2_, and were passaged using 0.25% EDTA containing trypsin (Mediatech Inc). A hemocytometer was used to determine cell number. Peptide synthesis reagents were obtained from either Anaspec or AAPPTEC with the highest grade available (>99% purity) and used without further purification. Solvents and other chemical reagents were obtained from Sigma-Aldrich, unless otherwise stated.

### Selection of peptide specific for FGFR2

We performed peptide selection using the extracellular domain (ECD) of FGFR2c. This region of the target is accessible to imaging. We obtained recombinant FGFR2-ECD (Met1-Glu377) consisting of 367 amino acids after removal of the signal peptide (#10824-H08H-50, Sino Biological). We performed SDS-PAGE with 1 μg of FGFR2-ECD to evaluate the quality and quantity using 0.25, 0.5, and 1 μg of BSA as control. Peptide selection was performed using a phage display library (New England Biolabs, Ph.D.-12) per manufacturer instructions. This library consists of M13 bacteriophage that expresses ~10^9^ unique 12-amino acid sequences. 2×10^11^ pfu consisting of 2×10^9^ unique clones with ~100 copies each were biopanned against FGFR2-ECD immobilized in a 6-well plate at 4°C. 4 rounds of biopanning were performed using a decreasing quantity (100, 80, 60, and 40 μg) of FGFR2-ECD in successive rounds to increase binding specificity. After the 4th round, 50 plaques were randomly selected for DNA preparation and sequence analysis. We used an ABI Automatic DNA Analyzer (Applied Biosystems) with primer 5′-CCCTCATAG TTA GCG TAA CG-3′ (−96 gIII sequencing primer, New England Biolabs) that corresponds to the pIII gene sequence of the M13 phage.

### Synthesis of peptide specific for FGFR2

We used standard Fmoc-mediated solid-phase synthesis to produce the Cy5.5-labeled peptides [45]. We assembled Fmoc and Boc protected *L*-amino acids on rink amide MBHA resin. The peptides were synthesized using a PS3 automatic synthesizer (Protein Technologies Inc). The C-terminal lysine was incorporated as Fmoc-Lys (ivDde)-OH, and the N-terminal amino acid was incorporated with Boc protection to avoid unwanted Fmoc removal during deprotection of the ivDde moiety prior to fluorophore labeling. Upon complete assembly of the peptide, the resin was transferred to a reaction vessel for manual labeling with dye. The ivDde side chain protecting group was removed with 5% hydrazine in DMF (3×10 min) with continuous shaking at room temperature (RT). The resin was washed with dimethylformamide (DMF) and dichloromethane (DCM) 3X each for 1 min. The protected resin-bound peptide was incubated overnight with Cy5.5-NHS ester (Lumiprobe LLC) with DIEA, and the completion of the reaction was monitored by a qualitative Ninhydrin test. Upon completion of labeling, the peptide was cleaved from the resin using TFA:TIS:H2O (95:2.5:2.5 v/v/v; Sigma-Aldrich) for 4 hours with shaking in the dark at RT. After separating the peptide from the resin, the filtrate was evaporated with N_2_ gas followed by precipitation with chilled diethyl ether and stored overnight at −20°C. The precipitate was centrifuged at 3000 rpm for 5 min and washed with diethyl ether 3X and centrifuged in between each washing step. The crude peptides were dissolved in 1:1 acetonitrile/H2O (v/v) and purified by prep-HPLC with a C_18_ column (Waters Inc) using a water (0.1% TFA)-acetonitrile (0.1% TFA) gradient. The final purity of the peptides was confirmed with an analytical C_18_-column. Further characterization was performed with either ESI (Waters Inc) or Q-TOF (Agilent Technologies) mass spectrometry.

### Confocal fluorescence microscopy

~10^3^ QhTERT/wt, QhTERT/FGFR2b, and QhTERT/FGFR2c cells were grown on cover glass to ~80% confluence. The cells were washed with PBS 1X and incubated with 1 μM of either peptide for 10 min at RT. The cells were then washed 3X in PBS, fixed with 4% paraformaldehyde (PFA) for 5 min, washed 1X with PBS, and then mounted on glass slides with ProLong Gold reagent containing DAPI (Invitrogen). Confocal fluorescence images were collected with Cy5.5 and DAPI filters (Leica Inverted SP5X 2-Photon FLIM confocal microscopes) using a 63X oil-immersion objective. Fluorescence intensities from 3 cells in each of 3 independent images were quantified using custom Matlab (Mathworks) software.

### Competition for peptide binding

Specific peptide binding to QhTERT/FGFR2c was validated on competitive inhibition with addition of unlabeled peptide. ~10^3^ cells were grown to ~70% confluence on cover glass in triplicate. Unlabeled peptides at concentrations of 0, 50, 100, 150, 250, and 500 μM were incubated with the cells for 30 min at 4°C. The cells were washed and incubated with 5 μM of the target peptide for another 30 min at 4°C. The cells were washed and fixed with 4% PFA for 5 min. The cells were washed with PBS and mounted with ProLong Gold reagent containing DAPI (Invitrogen).

### Characterization of peptide binding

We measured the apparent dissociation constant k_d_ for peptide binding to cells to assess binding affinity [46]. The Cy5.5-labeled peptide was serially diluted in PBS at concentrations ranging from 0 to 200 nM in 25 nM increments. QhTERT/FGFR2c cells (~10^5^) were incubated with peptide at 4°C for 1 hour, washed with cold PBS, and the mean fluorescence intensities were measured using flow cytometry. The equilibrium dissociation constant k_d_=1/k_a_ was calculated by performing a least squares fit of the data to the non-linear equation I=(I_0_+I_max_k_a_[X])/(I_0_+k_a_[X]). I_0_ and I_max_ are the initial and maximum fluorescence intensities, corresponding to no peptide and at saturation, respectively, and [X] represents the concentration of the bound peptide. Prism 5.0 software (GraphPad Inc) was used to calculate k_d_.

We measured the apparent association time constant of the peptide to QhTERT/FGFR2c cells to assess binding onset [47]. Cells were grown to ~80% confluence in 10 cm dishes, and detached with PBS-based cell dissociation buffer (Invitrogen). Cells (~10^5^ were incubated with 5 μM SRR-Cy5.5 at RT for various time intervals ranging from 0 to 30 min. The cells were centrifuged, and washed with cold PBS. Flow cytometry analysis was performed as described above, and the median fluorescence intensity (y) were measured on flow cytometry at different time points (t) using Flowjo software. The rate constant k was calculated by fitting the data to a first order kinetics model, y(t) = I_max_[1-exp^(-kt)^], where I_max_ = maximum value using Prism 5.0 software (GraphPad Inc).

### Binding of FGFR2 peptide and antibody to human esophageal specimens

Formalin-fixed sections of human esophageal specimens were deparaffinized, and antigen retrieval was performed using standard methods. Briefly, the sections were incubated in xylene for 3 min 3X, washed with 100% ethanol for 2 min 2X, and washed with 95% ethanol for 2 min 2X. Rehydration was performed by washing in dH_2_O for 5 min 2X. Antigen unmasking was performed by boiling the slides in 10 mM sodium citrate buffer with 0.05% Tween at pH 6.0, and then maintaining at sub-boiling temperature for 15 min. The slides were cooled for 30 min, and the sections were washed in dH_2_O for 3 min 3X and in PBS for 5 min. Blocking was performed with DAKO protein blocking agent (X0909, DAKO) for 1 hour at RT. The peptides were incubated at a concentration of 1 μM for 10 min at RT. The sections were washed in PBS for 3 min 3X, and incubated with 1:1000 dilution of monoclonal anti-FGFR2 (Abcam, ab58201) overnight at 4°C.

The sections were then washed in PBS for 5 min 3X. A 1:500 dilution of AF488-labeled secondary antibody (goat anti-mouse) was added to each section and incubated for 30 min at RT. The secondary antibody solution was removed by washing with PBS for 5 min 3X. The sections were then mounted with ProLong Gold reagent containing DAPI (Invitrogen). The mean fluorescence intensities from 3 boxes (dimensions of 30×30 μm^2^) located completely within the surface epithelium of each specimen were measured. Regions that showed intensity saturation were avoided. Serial sections were processed for routine histology (H&E), and were reviewed by an expert gastrointestinal pathologist (HDA).

### Effect of peptide on cell signaling

QhTERT cells that overexpress either FGFR2b or FGFR2c were seeded in 12-well flat-bottom plates with 500 μL of serum-free medium for 16 hours. FGF1 (#2232-FA-025, R&D systems) was reconstituted to a concentration of 100 μg/mL using PBS, diluted with 0.1% bovine serum albumin, and added to the cells at final concentrations of 100 ng/mL for 20 min in separate wells. Heparin (# H3149-10KU, Sigma) with final concentration of 100 units/mL was also added to increase stability. In addition, peptides at concentrations of 5 and 100 μM were incubated for 20 min in separate wells. The cells were washed with PBS and lysed in RIPA buffer containing protease inhibitors (#11836170001, Roche, Basel, Switzerland). Lysates were separated by gel electrophoresis, transferred to polyvinylidene difluoride membranes (#ISEQ00010, Millipore), and detected by immunoblotting using an enhanced chemiluminescence system (#RPN2106, GE Healthcare). Anti-FGFR2 antibody (#SC122, Santa Cruz Biotechnology), anti-phospho-FGFR (#3471, Cell Signaling Technology), anti-AKT (#4691P, Cell Signaling Technology), anti-ERK1/2 (#4695P, Cell Signaling Technology), anti-phospho-AKT (pS473; #4060P, Cell Signaling Technology), anti-phospho-ERK1/2 (#4370P, Cell Signaling Technology), and anti-tubulin (#32–2600, Invitrogen) were used as per manufacturer's instructions.

## SUPPLEMENTARY MATERIALS FIGURES



## Abbreviations

BE: Barrett's esophagus
EAC: esophageal adenocarcinoma
EMR: endoscopic mucosal resection
FGFR: fibroblast growth factor receptor
HGD: high-grade dysplasia
IF: immunofluorescence
IHC: immunohistochemistry
LGD: low-grade dysplasia
NIR: near-infrared
ROI: regions of interest
RFA: radio-frequency ablation
RT: room temperature
RTK: receptor tyrosine kinase
SQ: squamous
T/B: target-to-background
