# Supplementary material for: Identification and validation of FGFR2 peptide for detection of early Barrett's neoplasia
Source: Oncotarget. 2017 Aug 1;8(50):87095–106. doi: 10.18632/oncotarget.19764 (PMC5675618; doi:10.18632/oncotarget.19764)
Supplement: Supplementary file 1 [file oncotarget-08-87095-s001.pdf]

Identification and validation of FGFR2 peptide for detection of early Barrett's neoplasia

SUPPLEMENTARY MATERIALS

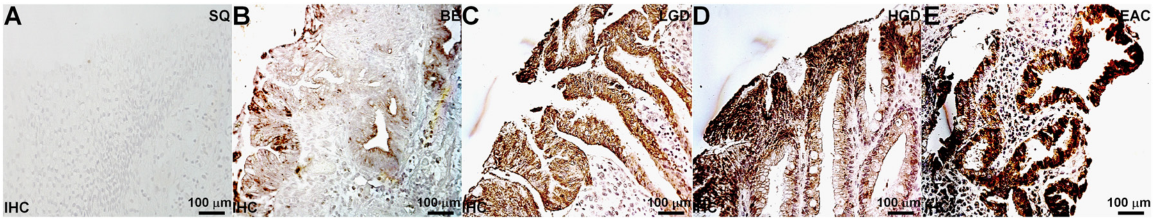

Supplementary Figure 1: Immunohistochemistry (IHC) of Barrett's neoplasia. Representative IHC from sections of human esophagus ex vivo shows increasing expression of FGFR2 with histological progression from **(A)** squamous (SQ), **(B)** Barrett's esophagus (BE), **(C)** low-grade dysplasia (LGD), **(D)** high-grade dysplasia (HGD), and **(E)** esophageal adenocarcinoma (EAC).

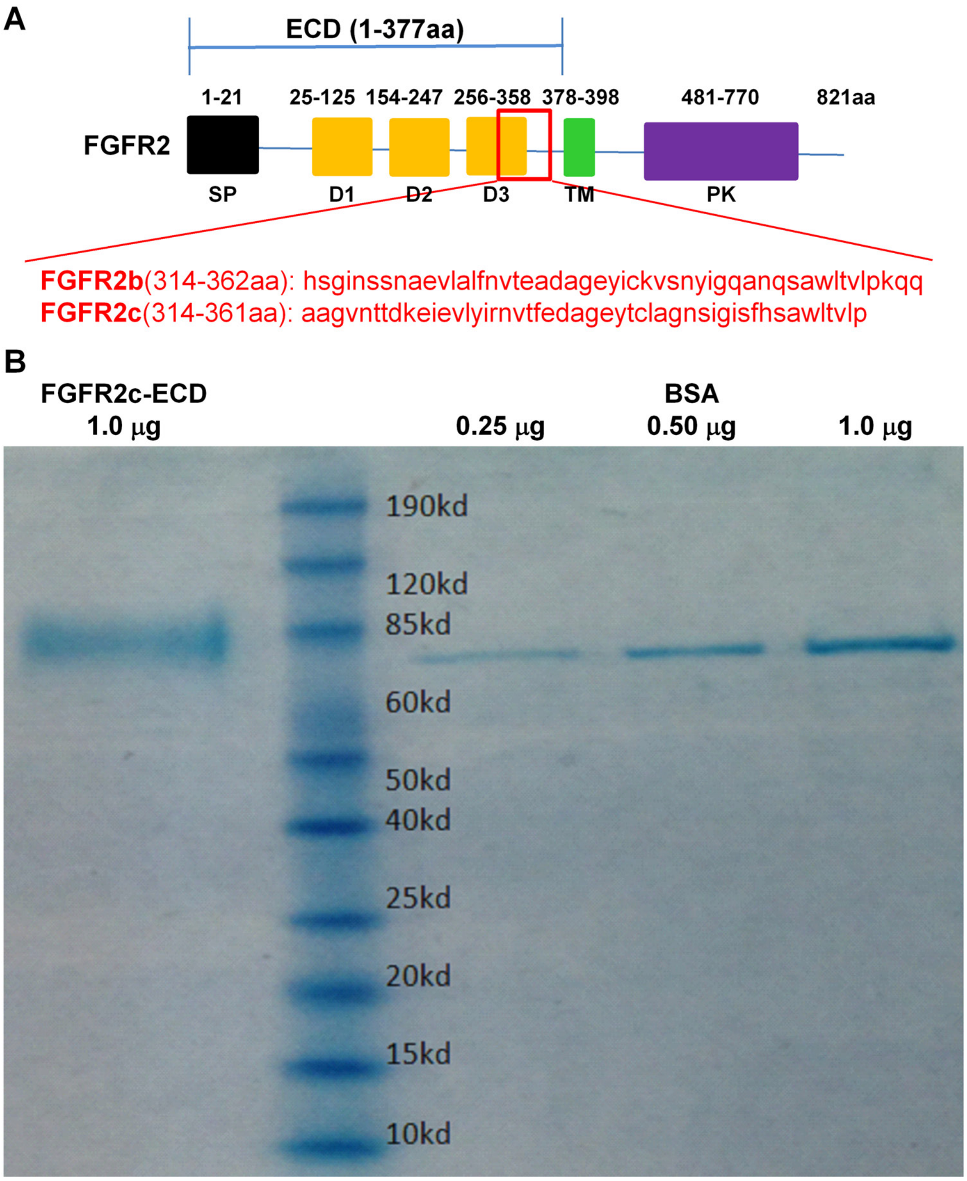

Supplementary Figure 2: Characterization of FGFR extracellular domain (ECD). (A) Schematic diagram shows that FGFR2-ECD contains a signal peptide (SP) and 3 extracellular immunoglobulin-like domains (D1-D3). FGFR2-ECD is anchored by a hydrophobic transmembrane region (TM) to the cytoplasmic domain that contains a tyrosine kinase catalytic domain (PK). Alternative splicing of FGFR2 in either exon 8 or 9 results in expression of either FGFR2b or FGFR2c, respectively, in the C-terminus of D3. The differences in amino acids 314-362 is shown (red). **(B)** For recombinant FGFR2-ECD, we achieved a purity >97% by HPLC. Using SDS-PAGE, we observed an apparent molecular mass of ~65-75 kDa that reflects glycosylation. Standard BSA is shown for comparison.

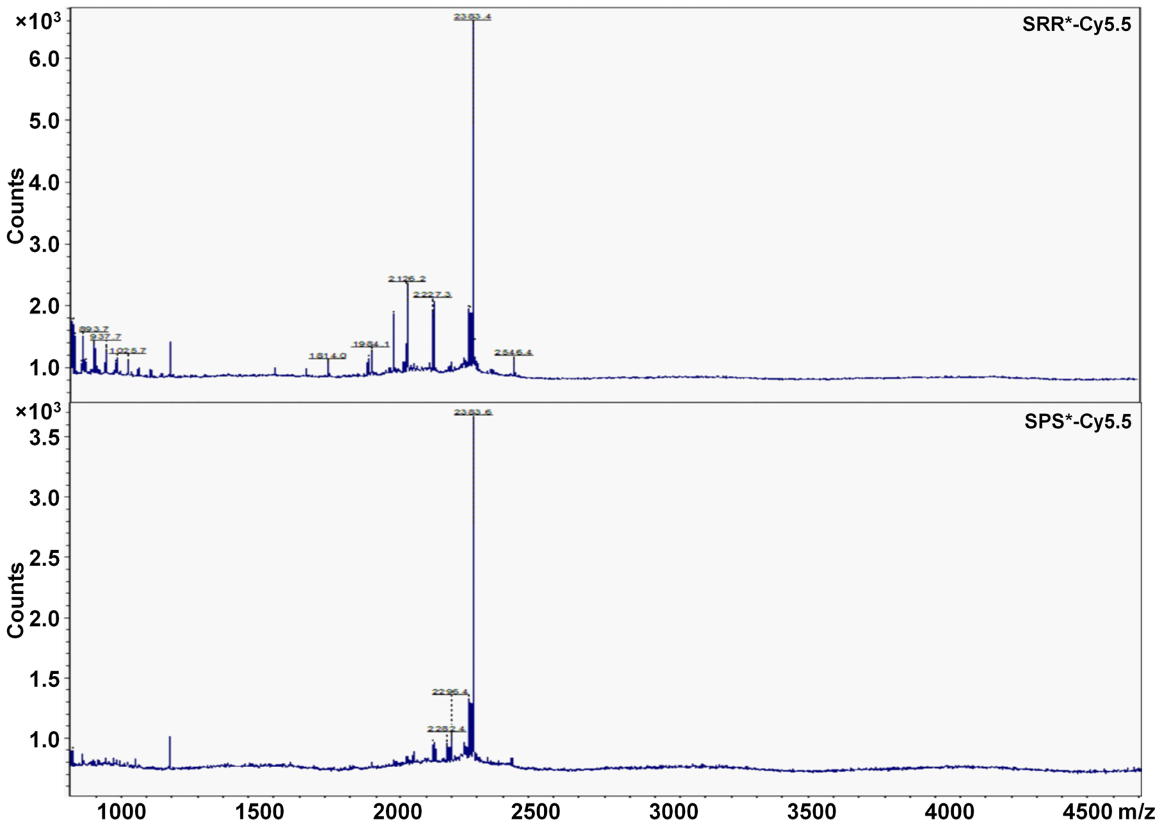

Supplementary Figure 3: Mass spec of FGFR2 peptides. We measured an experimental mass-to-charge (m/z) ratio of 2385.31 for both SRR*-Cy5.5 and SPS*-Cy5.5. These results agree with expected values.

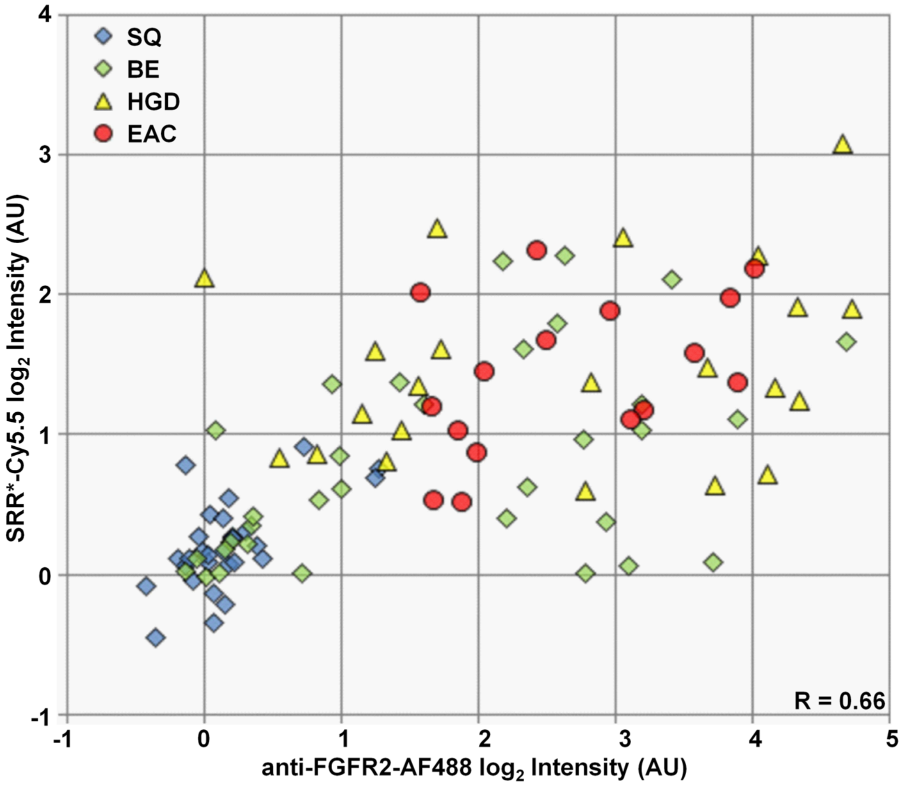

Supplementary Figure 4: Correlation between FGFR2 peptide and antibody. Using immunofluorescence, we found good correlation between SRR*-Cy5.5 peptide and AF488-labeled anti-FGFR2 antibody. We measured an overall Pearson's coefficient of ρ = 0.66, $P = 1.4 \times 10^{-13}$ for staining of n = 28, 33, 22, and 17 human esophageal specimens of SQ, BE, HGD, and EAC, respectively.

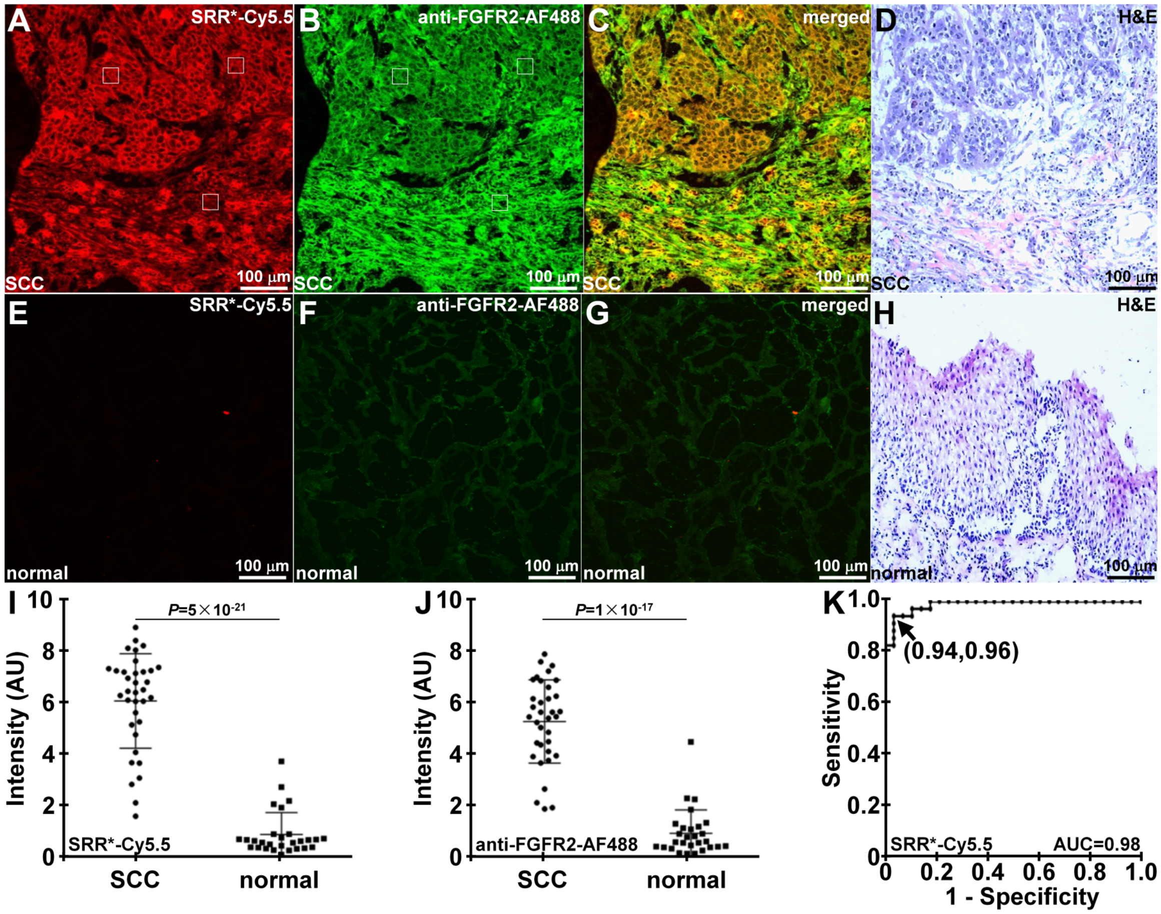

Supplementary Figure 5: Binding of FGFR2 peptide to human esophageal squamous cell carcinoma (SCC). On representative images collected with confocal microscopy, **(A)** SRR*-Cy5.5 (red) and **(B)** anti-FGFR2 antibody labeled with AF488 (green) shows strong binding to sections of human esophageal SCC. **(C)** Pearson's correlation coefficient of $\rho = 0.84$ was found on merged image. **(D)** Corresponding histology (H&E) for SCC. By comparison, we found minimal staining with **(E)** SRR*-Cy5.5 and **(F)** AF488-labeled anti-FGFR2 antibody to sections of normal human esophagus. **(G)** Merged image. **(H)** Corresponding histology (H&E) of normal esophagus. We quantified the fluorescence intensities from the mean of a set of 3 boxes with dimensions of 30×30 μm^2 placed randomly, shown in panels (A) and (B). We found significantly greater mean fluorescence intensity from SCC versus normal for **(I)** SRR*-Cy5.5, $P=5\times10^{-21}$ and **(J)** AF488-labeled anti-FGFR2 antibody, $P=1\times10^{-17}$, by paired, two-sided t-test. **(K)** ROC curve shows 94% sensitivity and 96% specificity for detecting SCC.

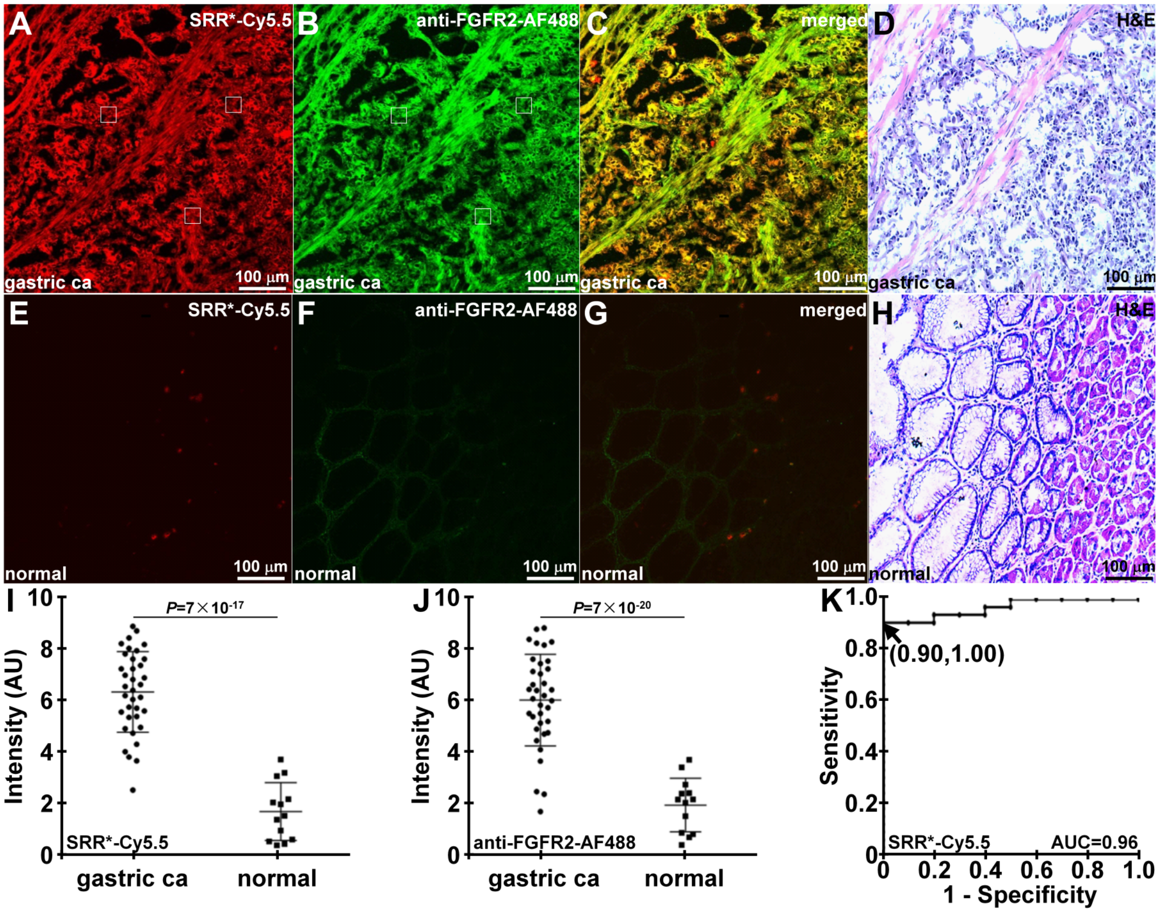

Supplementary Figure 6: Binding of FGFR2 peptide to human gastric cancer. On representative images collected with confocal microscopy, **(A)** SRR*-Cy5.5 (red) and **(B)** anti-FGFR2 antibody labeled with AF488 (green) shows strong binding to sections of human gastric cancer. **(C)** Pearson's correlation coefficient of $\rho = 0.93$ was found on merged image. **(D)** Corresponding histology (H&E) for gastric cancer. By comparison, we found minimal staining with **(E)** SRR*-Cy5.5 and **(F)** AF488-labeled anti-FGFR2 antibody to sections of normal human stomach. **(G)** Merged image. **(H)** Corresponding histology (H&E) of normal stomach. We quantified the fluorescence intensities from the mean of a set of 3 boxes with dimensions of 30×30 μm^2 placed randomly, shown in panels (A) and (B). We found significantly greater mean fluorescence intensity from gastric cancer versus normal for **(I)** SRR*-Cy5.5, $P=7\times10^{-17}$ and **(J)** AF488-labeled anti-FGFR2 antibody, $P=7\times10^{-20}$, by paired, two-sided t-test. **(K)** ROC curve shows 90% sensitivity and 100% specificity for detecting gastric cancer.
